# A comprehensive atlas of pig RNA editome across 23 tissues reveals RNA editing affecting interaction mRNA–miRNAs

**DOI:** 10.1093/g3journal/jkae178

**Published:** 2024-08-02

**Authors:** Jiajia Long, Weiwei Liu, Xinhao Fan, Yalan Yang, Xiaogan Yang, Zhonglin Tang

**Affiliations:** Guangxi Key Laboratory of Animal Breeding, Disease Control and Prevention, College of Animal Science & Technology, Guangxi University, Nanning, Guangxi 530004, China; Kunpeng Institute of Modern Agriculture at Foshan, Agricultural Genomics Institute, Chinese Academy of Agricultural Sciences, Foshan 528226, China; Shenzhen Branch, Guangdong Laboratory for Lingnan Modern Agriculture, Key Laboratory of Livestock and Poultry Multi-Omics of MARA, Agricultural Genomics Institute at Shenzhen, Chinese Academy of Agricultural Sciences, Shenzhen 518124, China; Guangxi Key Laboratory of Animal Breeding, Disease Control and Prevention, College of Animal Science & Technology, Guangxi University, Nanning, Guangxi 530004, China; Kunpeng Institute of Modern Agriculture at Foshan, Agricultural Genomics Institute, Chinese Academy of Agricultural Sciences, Foshan 528226, China; Shenzhen Branch, Guangdong Laboratory for Lingnan Modern Agriculture, Key Laboratory of Livestock and Poultry Multi-Omics of MARA, Agricultural Genomics Institute at Shenzhen, Chinese Academy of Agricultural Sciences, Shenzhen 518124, China; Kunpeng Institute of Modern Agriculture at Foshan, Agricultural Genomics Institute, Chinese Academy of Agricultural Sciences, Foshan 528226, China; Shenzhen Branch, Guangdong Laboratory for Lingnan Modern Agriculture, Key Laboratory of Livestock and Poultry Multi-Omics of MARA, Agricultural Genomics Institute at Shenzhen, Chinese Academy of Agricultural Sciences, Shenzhen 518124, China; Key Laboratory of Agricultural Animal Genetics, Breeding and Reproduction of Ministry of Education & Key Lab of Swine Genetics and Breeding of Ministry of Agriculture and Rural Affairs, Huazhong Agricultural University, Wuhan 430070, China; Shenzhen Branch, Guangdong Laboratory for Lingnan Modern Agriculture, Key Laboratory of Livestock and Poultry Multi-Omics of MARA, Agricultural Genomics Institute at Shenzhen, Chinese Academy of Agricultural Sciences, Shenzhen 518124, China; Guangxi Key Laboratory of Animal Breeding, Disease Control and Prevention, College of Animal Science & Technology, Guangxi University, Nanning, Guangxi 530004, China; Guangxi Key Laboratory of Animal Breeding, Disease Control and Prevention, College of Animal Science & Technology, Guangxi University, Nanning, Guangxi 530004, China; Kunpeng Institute of Modern Agriculture at Foshan, Agricultural Genomics Institute, Chinese Academy of Agricultural Sciences, Foshan 528226, China; Shenzhen Branch, Guangdong Laboratory for Lingnan Modern Agriculture, Key Laboratory of Livestock and Poultry Multi-Omics of MARA, Agricultural Genomics Institute at Shenzhen, Chinese Academy of Agricultural Sciences, Shenzhen 518124, China; Key Laboratory of Agricultural Animal Genetics, Breeding and Reproduction of Ministry of Education & Key Lab of Swine Genetics and Breeding of Ministry of Agriculture and Rural Affairs, Huazhong Agricultural University, Wuhan 430070, China

**Keywords:** pig, RNA editing, multiple tissue, skeletal muscle, miRNA

## Abstract

RNA editing is a co-transcriptional/post-transcriptional modification that is mediated by the ADAR enzyme family. Profiling of RNA editing is very limited in pigs. In this study, we collated 3813 RNA-seq data from the public repositories across 23 tissues and carried out comprehensive profiling of RNA editing in pigs. In total, 127,927 A-to-I RNA-editing sites were detected. Our analysis showed that 98.2% of RNA-editing sites were located within repeat regions, primarily within the pig-specific SINE retrotransposon PRE-1/Pre0_SS elements. Subsequently, we focused on analyzing specific RNA-editing sites (SESs) in skeletal muscle tissues. Functional enrichment analyses suggested that they were enriched in signaling pathways associated with muscle cell differentiation, including DMD, MYOD1, and CAV1 genes. Furthermore, we discovered that RNA editing event in the 3′UTR of CFLAR mRNA influenced miR-708-5p binding in this region. In this study, the panoramic RNA-editing landscape of different tissues of pigs was systematically mapped, and RNA-editing sites and genes involved in muscle cell differentiation were identified. In summary, we identified modifications to pig RNA-editing sites and provided candidate targets for further validation.

## Introduction

RNA editing is one of the ways to modify RNA post-transcriptionally or co-transcriptionally that increases the diversity of subsequent transcripts and proteins by changing nucleotide in the RNA sequence (Yang *et al*. 2019; Gabay *et al*. 2022). In mammals, the most common type of RNA editing is adenosine (A) to adenosine (I) RNA editing (AIRESs), which is catalyzed by the ADAR enzyme family to deaminate adenosine to inosine. This family had 3 members that act on the mammalian genome, namely, ADAR1, ADAR2, and ADAR3 (Jimeno *et al*. 2021; Shiromoto *et al*. 2021; Teoh *et al*. 2021). Inosine was recognized as guanosine (G) by cellular mechanisms during reverse transcription and translation, so A-to-I RNA editing was also known as A-to-G RNA editing (Jiang and Zhang 2019). A-to-I RNA editing in the protein-coding region (CDS) may change the result of translation, thereby affecting other biological processes (Yang *et al*. 2019; Schaffer *et al*. 2020; Ma *et al*. 2021). A-to-I RNA editing in noncoding regions usually affected alternative splicing (Wu *et al*. 2020; Noda *et al*. 2022; Lebrigand *et al*. 2023), miRNA binding to mRNA (Ramírez-Moya *et al*. 2020; Voss *et al*. 2023), and the generation of circular RNA (circRNA) (Heraud-Farlow and Walkley 2020; Shen *et al*. 2022).

The pig (*Sus scrofa*) is an important livestock animal and accounts for over one-third of human meat consumption (Xu *et al*. 2023; Zhou *et al*. 2023) and the domestication of pigs has a long history (Ling *et al*. 2024). A-to-I RNA editing is crucial for normal vertebrate development. A recent study established a comprehensive and dynamic map of the skeletal muscle editome across 27 developmental stages of pig skeletal muscle, providing new insights into muscle development (Yang *et al*. 2019). Research on RNA editing in pigs could facilitate our exploration of porcine genomic variation mechanisms at the post-transcriptional level, providing potential targets for pig molecular breeding. Recent studies have shown RNA-editing events in pig could prevent the miRNA-mediated mRNA downregulation of HSPA12B in the muscle-derived satellite (MDS) cell (Adetula *et al*. 2021) and play a key role in transcriptional regulatory machinery in the porcine endometrium in response to lipopolysaccharide treatment (Paukszto *et al*. 2020). These results highlight the importance of A-to-I RNA editing in pig genetic breeding.

In recent years, with the rapid development of high throughput sequencing technology, a large amount of RNA-seq data has been generated, and studies on RNA editing events in different tissues or breeds of pigs have emerged (Wang *et al*. 2019, 2020; Yang *et al*. 2019, 2023; Li *et al*. 2020). However, existing studies were limited in terms of sample quantity and have not utilized large-scale data to dissect the A-to-I RNA-editing landscape and editing patterns across different pig tissues. To better utilize large-scale RNA-seq data, we collected a total of 3,813 RNA-seq datasets and performed a comprehensive profiling of RNA editing in pigs. Here, we detected a total of 135,246 RNA editing sites in all samples. In addition, we conducted molecular feature analysis on A-to-I RNA editing sites (AIRESs) and identified RNA-editing sites and genes related to important economic traits such as muscle development.

## Materials and methods

### Collection of RNA-seq data and identification of RNA-editing sites

All RNA-seq data of pigs were downloaded from the Sequence Read Archive (SRA) database of the National Center for Biotechnology Information (NCBI) and the China Nucleotide Sequence Archive (CNSA), information about all RNA-seq data across 23 tissues could be found in Supplementary Table 1. The reference genome file (Sus_scrofa.Sscrofa11.1.dna.toplevel.fa) and annotation file (Sus_scrofa.Sscrofa11.1.111.gtf) of *Sus scrofa* (v11.1) were downloaded from the Ensembl database. Clean data reads were aligned to the reference genome using HISAT2 (v1.6). The view command of samtools (v1.6) was used to convert Sequence Alignment/Map Format (SAM) files to Binary Alignment/Map Format (BAM) files, and the sort command was used to sort BAM files. The “sprint_from_bam” command of SPRINT (v0.1.8) was used to identify RNA-editing sites from the sorted BAM files (https://github.com/jumphone/SPRINT). To improve the accuracy of RNA-editing sites, the following screening was performed on each file generated by the SPRINT software using R software (v4.3.2): (1) the site had at least 10 RNA reads coverage; (2) at least 3 reads of the site could be detected by the SPRINT software; (3) the editing rate of the site were at least 0.05; (4) all known single nucleotide polymorphism (SNP) sites were filtered using the pig dbSNP database (https://ftp.ensembl.org/pub/release-109/variation/vcf/sus_scrofa/); (5) according to the method for identifying the major sources of variation in tissue samples (Tan *et al*. 2017), we performed PCA with Rstne package(v0.17) of R software (Supplementary Fig. 1), and removed sites in each tissue (Supplementary Table 2).

Information about all RNA-seq data, such as SRR ID, project, source, mapping rate, read length and read type, was documented in Supplementary Table 1. Additionally, we verified that the datasets used were not under any embargo or data sharing agreement at the time of use.

### Validation of RNA-editing sites

In order to identify RNA-editing events, 17 AIRESs were randomly selected for verification by Sanger sequencing. Two samples (CNR0262766 and CNR0262778) used for Sanger sequencing were obtained from our previous report and are available from the China National GenBank Nucleotide Sequence Archive (CNSA) under accession number CNP0001159 (https://db.cngb.org/search/project/CNP0001159/). Total DNA was extracted using HiScript III 1st Strand cDNA Synthesis Kit (+gDNA wiper) (TIANGEN BIOTECH, Beijing, China) according to the manufacturer's protocols. Total RNA was extracted by TRIzol Reagent and reverse-transcribed into cDNA using the HiScript^®^ III 1st Strand cDNA Synthesis Kit (+gDNA wiper) (Vazyme, Nanjing, China) according to the manufacturer's protocols. The selected regions were amplified from the gDNA and cDNA samples by the primers listed in Supplementary Table 3. As mentioned above (Yang *et al*. 2019), the PCR products were sent to Sanger sequencing (Sangon Biotech, Shanghai, China).

### Data analysis of RNA-editing sites

In this study, we focused on A-to-I RNA-editing sites, and all RNA-editing sites used in subsequent analyses are A-to-I editing. Annotation of pig genes was performed using the Ensembl online annotation tool (http://www.ensembl.org/Multi/Tools/VEP). The pig repeat region file (rmsk.txt) was downloaded from the University of California Santa Cruz (UCSC) database, and the intersectBed command of bedtools (v2.3.0) was used to compare the results of all sites; rmsk.txt was performed to obtain the annotation results of repeat region of all sites. bedtools was used to extract the sequences of all AIRESs and their upstream and downstream 5 bp bases from the reference genome file for analysis of base preference. To determine expression level of the ADAR enzyme, we used the bam file of RNA-seq data to calculate the transcript per million (TPM) value for quantification through StringTie (v2.2.1) and the limma R package (v3.54.0) was used to eliminate the batch effect of samples of different tissues. Mature miRNA sequences were downloaded from miRbase (release 22.1), and miRanda (v3.3a) (https://bioweb.Pasteur.fr/packages/pack@miRanda@3.3a) was used to predict miRNAs and 2 sequences (upstream and downstream 150 bp) that occurred in the 3′ untranslated region (UTR) of the gene with and without RNA editing.

### Analysis of tissues-specific RNA-editing sites

To detect SESs, all of the available data for just 7 tissues (brain, liver, adipose, intestine, heart, skeletal muscle, and lung) were selected from the wider downloaded RNA-Seq dataset (following the screening described in ‘*Collection of RNA-sseq data and identification of RNA-editing sites*’). Before this step, we removed sites in each tissue (see ‘*Collection of RNA-sseq data and identification of RNA-editing sites*’). The ROKU function of the TCC package (v1.20.1) of R software was utilized to rank the editing sites based on their overall tissue-specificity using Shannon entropy and to identify the tissue-specially editing sites, all SESs required Shannon entropy lower than 0.5. Gene Ontology (GO) analysis was performed using the Annotation, Visualization, and Integrated Discovery (DAVID) website (v2021, https://david.ncifcrf.gov/) (Sherman *et al*. 2022).

### Vector construction

Using RNA and DNA templates from CNR0262766, which were obtained from our previous report and are available from the China National GenBank Nucleotide Sequence Archive (CNSA) under accession number CNP0001159 (https://db.cngb.org/search/project/CNP0001159/), vectors were constructed, the primer information of vector construction in Supplementary Table 4. Specifically, designed primers were employed to amplify the sequence of the RNA-editing site within the CFLAR 3′UTR, which included the miR-708-5p seed region. This sequence included both the wild-type sequence, CFLAR-A (unmodified RNA sequence), and the experimental sequence, CFLAR-G (RNA sequence with editing, Supplementary Fig. 2). The RNA expression template was generated by annealing complementary oligonucleotides to form an RNA-encoding DNA sequence. The resulting annealed DNA fragments were ligated between the NheI and XhoI sites of the pmirGLO dual-luciferase vector. The DNA expression template was created by annealing DNA sequences and ligating the annealed DNA fragments between the NheI and XhoI sites of the pmirGLO dual-luciferase miRNA target expression vector obtained from Promega Corporation, Madison, WI, USA. Both RNA and DNA expression templates were independently introduced into DH5α competent cells (Tiangen). Transformation was followed by the confirmation of individual colonies through Sanger sequencing.

### Dual-luciferase reporter assay

To validate the relationship between miR-708-5p and the RNA-editing site within the seed region of the CFLAR 3′UTR, human embryonic kidney 293T (HEK293T) cells were cultured in Dulbecco's modified Eagle's medium (Gibco, CA, USA) supplemented with 10% fetal bovine serum (ExCell Bio, Uruguay) and 1% penicillin–streptomycin (Thermo Scientific, MA, USA). The cells were maintained in a humidified incubator at 37°C with 5% CO_2_. HEK293T cells at 60–70% confluence were transfected with CFLAR-A or CFLAR-G along with miR-708-5p mimics. Forty-eight hours after transfection, luciferase activity was quantified using the Dual-Luciferase Reporter Assay System Kit (Promega, WI, USA) on a GloMax 20/20 Luminometer (Promega).

## Results

### Systematic identification of RNA-editing sites

**Fig. 1.**
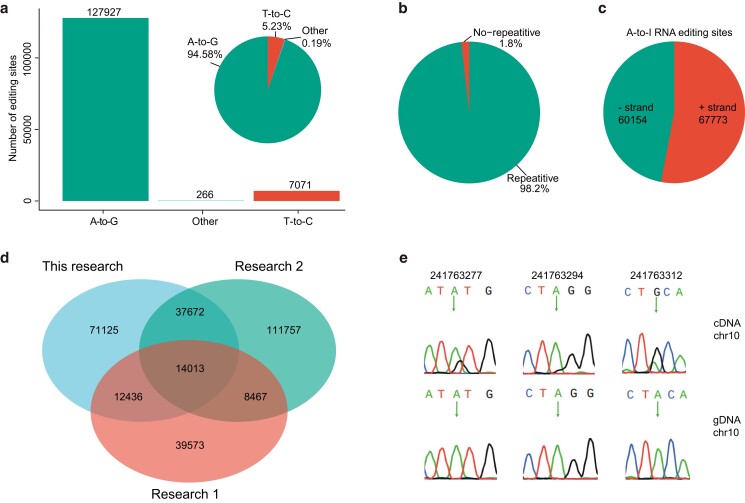
Identification of RNA-editing sites. a) The proportions and number of all types of RNA edits. b) Distribution of all RNA-editing sites in repetitive and nonrepetitive regions. c) A-to-I editing sites in reverse and forward strands. d) A Venn diagram comparing RNA-editing sites across this research, research 1 (Zhang *et al*. 2019), and research 2 (Adetula *et al*. 2021). e) Validation of A-to-I editing sites by Sanger sequencing, every candidate site was indicated by arrows.

To systematically map the panoramic RNA-editing landscape across 23 different tissues of pigs, we collected RNA-seq data from 3,813 samples (Supplementary Table 1). We detected a total of 135,246 RNA-editing sites (Supplementary Table 5), among which there were 2 main types of RNA editing. A-to-G conversion was the main type of RNA editing (127,927, 94.6%), consistent with previous reports in mammals (Adetula *et al*. 2021; Ma *et al*. 2021; Gabay *et al*. 2022; Zhang *et al*. 2022), followed by T-to-C conversion (7,071, 5.2%, Fig. 1a).

Among all the detected AIRESs, 60,154 and 67,773 sites were observed on the negative and positive chains, respectively (Fig. 1b), and most sites were located within the repeat region (125,639, 98.2%, Fig. 1c). As A-to-I editing was the most common type of editing, subsequent analyses mainly focus on A-to-I RNA type sites. We further compared RNA-editing sites of our research with 2 previously published studies (research 1 [Zhang *et al*. 2019], research 2 [Adetula *et al*. 2021]) and analyzed the RNA editome across tissues of pig.

To verify the accuracy of the RNA-editing sites identified in this study, we randomly selected 17 RNA-editing sites for experimental verification by PCR and Sanger sequencing. The results showed that 13 RNA-editing sites were successfully verified (Fig. 1f and Supplementary Fig. 3).

### Characterization of A-to-I RNA-editing sites

**Fig. 2.**
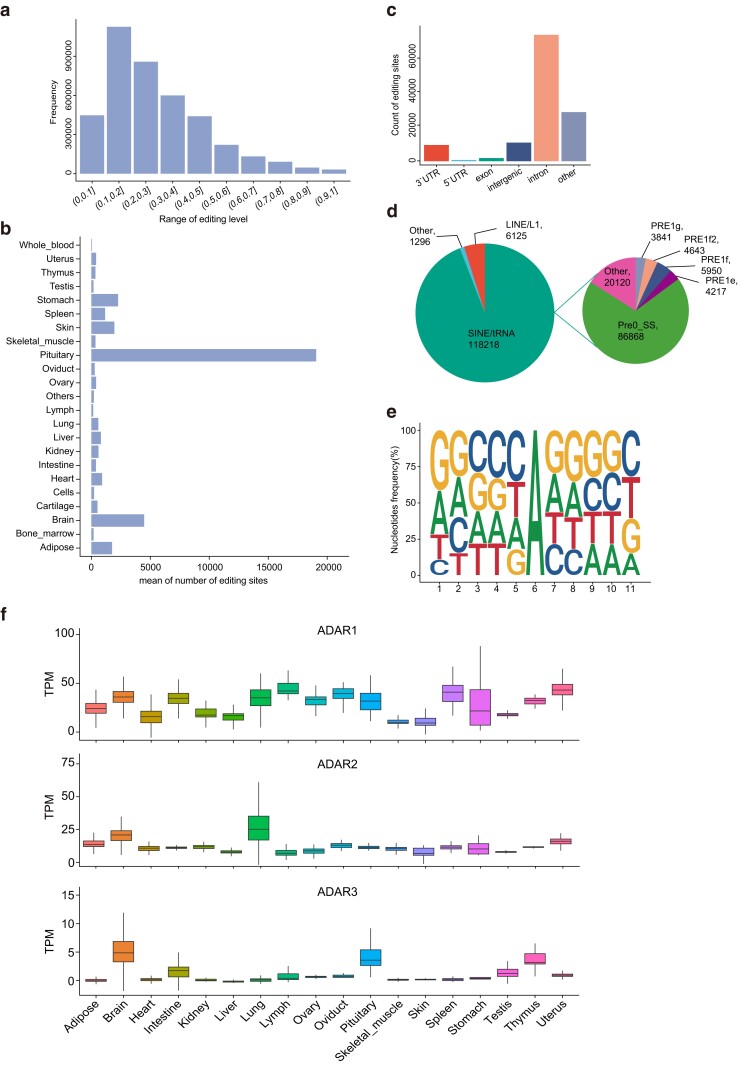
Molecular characterization of A-to-I RNA-editing sites in pigs. a) The distribution of the average editing level of all A-to-I editing sites. b) The average number of A-to-I editing sites in tissues. c) Number of A-to-I editing sites at different genomic regions. d) Number of A-to-I editing sites within repeat regions and elements. e) Nucleotide preference flanking the A-to-I editing sites. f) Expression levels of the ADAR1, ADAR2, and ADAR3 gene in different pig tissues.

We conducted statistical analysis of the A-to-I RNA editing in 23 tissues (Fig. 2a) and found that the overall editing level was relatively low, mainly between 10 and 20% (Fig. 2b).

Subsequently, we analyzed the characteristics of all AIRESs. Most AIRESs located in introns (75,371, 58.9%, Fig. 2c), and this result was consistent with previous studies in mammals (Gabay *et al*. 2022). In primates, RNA-editing sites were mostly located in Alu regions (Shiromoto *et al*. 2021; Rajendren *et al*. 2023), which was the most abundant subclass of short interspersed nuclear elements (SINEs) (Lu *et al*. 2021). Further analysis of the sites in the repeat region showed that 94.1% of AIRESs located in glutamic acid transfer RNA-derived SINEs (SINE/tRNA), followed by 4.9% in SINEs (LINE/L1) and 1% in other repeat elements, in the SINE/tRNA element, A-to-I editing sites were mostly distributed in Pre0_SS, followed by PRE1f, PRE1f2, PRE1e, and PRE1g (Fig. 2d). These results were consistent with previous studies (Huang *et al*. 2021; Nguyen *et al*. 2022).

The sequences upstream and downstream of AIRESs have a certain base preference, which can be used to evaluate the accuracy of the identified RNA editing sites (Huang *et al*. 2021; Dadush *et al*. 2024). To evaluate the base composition in the 5 bp regions surrounding all AIRESs, we computed nucleotide frequencies, and the ggseqlogo R package (Wagih 2017) was employed to visualize these nucleotide sequences. Notably, we found that the G base is enriched at the +1 position and has the lowest probability of occurrence at the −1 position (Fig. 2e), which is consistent with the characteristics previously reported in mammals (Yang *et al*. 2019; Rajendren *et al*. 2023; Dadush *et al*. 2024). These sequence preferences were considered as potential *cis*-regulatory mechanism of A-to-I RNA editing (Huang *et al*. 2021). In summary, these findings underscore the accuracy of our RNA editing site detection in this study.

Previous research showed that A-to-I RNA editing was catalyzed by the ADAR enzyme family (Zambrano-Mila *et al*. 2023). In this study, we investigated the expression levels of the ADAR enzyme family genes in all tissues (Supplementary Table 6). We used RNA-seq data to calculate the gene expression levels in all samples, and the limma R package was used to eliminate batch effects between tissue samples (following the screening described in ‘*Data analysis of RNA-editing sites*’). We found that the ADAR genes had higher expression levels in the brain, uterus, and lung, but had lower abundance in the skeletal muscle. ADAR1 had lower frequency levels in the skeletal muscle and skin; ADAR2 had higher concentration in the brain and lung; ADAR3 had higher density in the brain, pituitary, and thymus (Fig. 2f).

### Specificity analysis of A-to-I editing sites in skeletal muscle

**Fig. 3.**
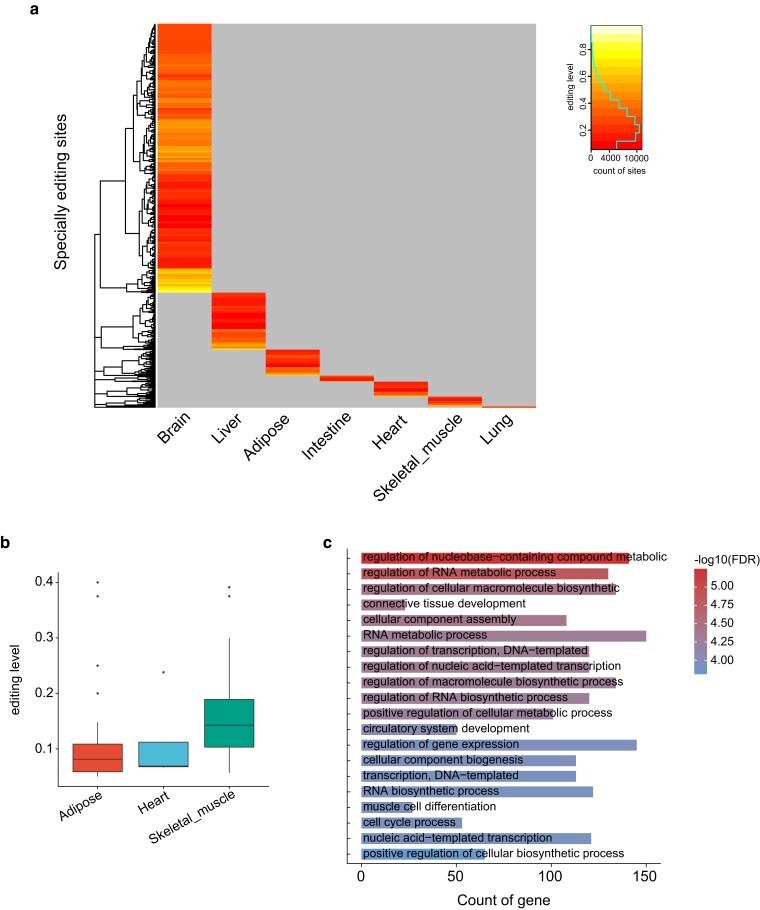
Tissue specific RNA-editing sites (SESs) in skeletal muscle and other tissues. a) Heat map of SESs in skeletal muscle and other tissues. b) The distribution of editing level with a randomly selected skeletal muscle-specific site across skeletal muscle and other tissues. c) The top 20 GO terms of all genes harboring SESs of skeletal muscle tissue.

SESs in the skeletal muscle and other tissues were identified (Fig. 3a and Supplementary Table 7). Following, we selected randomly a position among the skeletal muscle SESs (Fig. 3b). To explore the biological functions of genes harboring SESs in the skeletal muscle, we conducted gene enrichment analysis using GO terms (see ‘*Materials and methods*’). The results indicated that genes containing SESs in skeletal muscle significantly enriched in GO terms correlated with muscle cell differentiation (enrichment *P* < 0.05, Fig. 3c and Supplementary Table 8). Key genes in these pathways included the myogenic regulatory factor MYOD1 (Rovito *et al*. 2021; Martin *et al*. 2023), which influences muscle cell differentiation and muscle injury repair; DMD genes, which plays a role in skeletal muscle degeneration/regeneration (Hicks *et al*. 2023; Stec *et al*. 2023). These findings suggested that these specific-skeletal muscle SESs may impact myogenesis.

### RNA editing in the 3′UTR affects binding between CFLAR mRNA and miR-708-5p

**Fig. 4.**
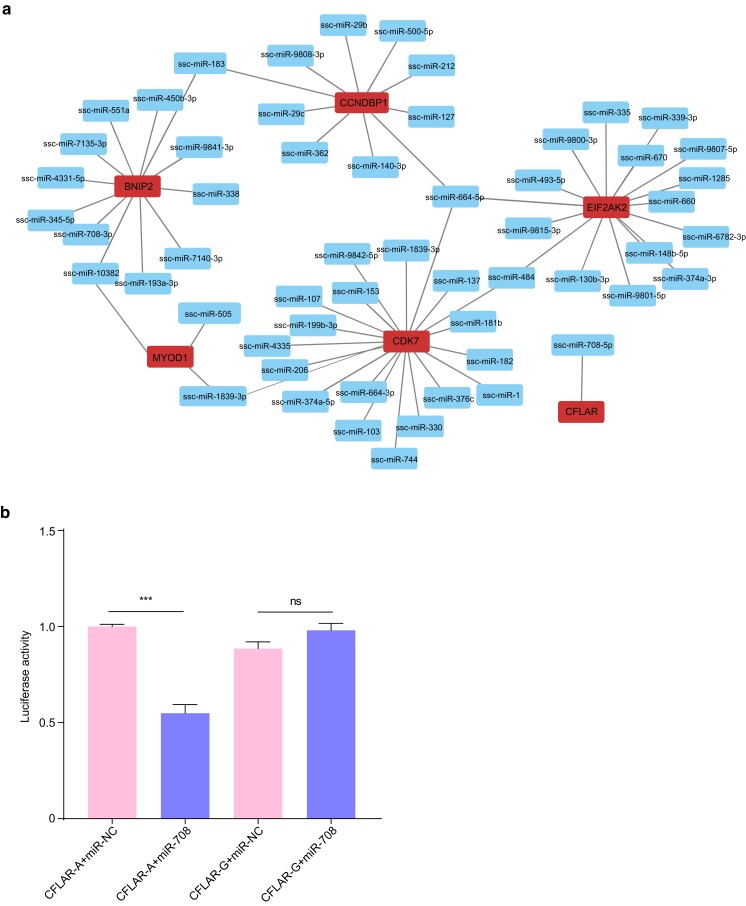
Impact of RNA editing on mRNA and miRNA interaction. a) Network miRNA and MYOD1, BNIP2, EIF2K2, CDK7, and CFLAR, and b) relative luciferase activity in HEK293T cells carrying edited CFLAR-G or not-edited CFLAR-A in the miR-708-5p miRNA-targeting regions. The *Y*-axis shows the relative luciferase activity. A 2-tailed Wilcoxon test was used to evaluate the difference in luciferase activity using R software, *n* = 3. Error bars signify standard errors. Significance in *b*): *P < 0.05; **P < 0.01; ***P < 0.001; ns, no significance.

RNA-editing sites within the 3′UTR of mRNA possess the capacity to exert an influence on the interaction between microRNA (miRNA) and mRNA, as previously explored in the literature (Ramírez-Moya *et al*. 2020; Adetula *et al*. 2021; Kim *et al*. 2023). A total of 11,713 A-to-I RNA editing sites were identified within the 3′UTR. From this pool, a subset of sites were strategically chosen from genes associated with skeletal muscle development (Ji *et al*. 2020; Ren and Cong 2021; Rovito *et al*. 2021; Tomaipitinca *et al*. 2021; Wang, Lu, *et al*. 2022). The prediction of miRNA target regions was performed using Miranda software (v3.3a). The obtained results delineate the intricate relationship between edited mRNAs and their corresponding target miRNAs (Fig. 4a and Supplementary Table 9).

CFLAR and miR-708-5p play pivotal roles in skeletal muscle development and repair (Tomaipitinca *et al*. 2021; Xu *et al*. 2022). Bioinformatic analysis revealed that RNA-editing events occurring in the miR-708-5p seed region of the 3′UTR of CFLAR mRNA could impact the binding of miR-708-5p to this region. To determine whether RNA editing in the 3′UTR affects the binding of miR-708-5p to CFLAR mRNA, 2 reporter plasmids carrying fluorescent enzyme sequences with edited and unedited CFLAR 3′UTRs were generated. The wild-type plasmid carried the unedited CFLAR 3′UTR sequence (CFLAR-A), while the experimental plasmid carried the edited CFLAR 3′UTR sequence (CFLAR-G); both contained the miR-708-5p seed region (the result of Sanger sequences, Supplementary Fig. 2). These plasmids were co-transfected with miR-708-5p into HEK293T cells, and luciferase activity was observed to assess the impact of miR-708-5p on the expression of the reporter gene with edited or unedited CFLAR 3′UTR.

The results suggested a significant decrease in luciferase activity when miR-708-5p was co-transfected with CFLAR-A into HEK293T cells, whereas there was no significant change when miR-708-5p was co-transfected with CFLAR-G into HEK293T cells. These experimental findings suggested that RNA editing of the miR-708-5p seed region in the CFLAR 3′UTR prevents the binding of miR-708-5p to CFLAR mRNA (Fig. 4b).

## Discussion

RNA editing, as a transcriptional/post-transcriptional modification, has been investigated based on its ubiquity and importance in regulating processes in mammals (Licht *et al*. 2019; Yang *et al*. 2019; Gabay *et al*. 2022; Wang, Ye *et al*. 2022). In recent years, with the rapid development of high throughput sequencing technology, many laboratories have studied RNA-editing events in pigs and other animals (Wang *et al*. 2020; Adetula *et al*. 2021; Huang *et al*. 2021; Larsen and Heide-Jørgensen 2021). However, the study on systematic identification and biological function of RNA editing events was limited in pigs. In this work, we firstly collected a total of 3,813 pig RNA-seq datasets, performed systematic identification, and analyzed molecular characteristics and function of RNA editing sites across different tissues in pigs.

We identified a total of 135,246 RNA-editing sites. The average distribution of sites in each sample in different tissues and the overall editing level was consistent with previous reports (Schaffer *et al*. 2020; Adetula *et al*. 2021; Gabay *et al*. 2022). AIRESs were the main type of editing and accounted for 94.6% of all editing sites. In addition, the upstream and downstream sequences of AIRESs had a certain base preference, which can be used to evaluate the accuracy of the detected RNA-editing sites. Our study showed that the 1 bp downstream of AIRESs had a strong preference for G, while the 1 bp upstream preference was the opposite (Fig. 2e). This preference was consistent with the known recognition sequence features of mammalian ADAR enzymes (Huang *et al*. 2021).

The majority of AIRESs identified in this study located in noncoding regions, such as introns and 3′UTR regions. However, noncoding sites do not directly change the result of translation. In recent years, the role of RNA editing in noncoding regions in gene regulation has been elucidated, with examples including RNA editing in the mRNA 3′UTR altering RNA stability, translation efficiency, and miRNA binding (Heraud-Farlow and Walkley 2020; Ramírez-Moya *et al*. 2020; Lebrigand *et al*. 2023).

In mammals, a significant proportion of AIRESs was primarily situated in repetitive elements such as SINEs. For instance, Alu elements in primates and PRE elements in pigs were prominent regions for RNA editing (Tan *et al*. 2017; Adetula *et al*. 2021). In humans, the ADAR enzyme family generally acts on double-stranded RNA (dsRNA) composed of reverse Alu repeat elements (Heraud-Farlow and Walkley 2020). While Alu elements were absent in the pig genome, studies indicated that PRE-1 elements were pig-specific SINE retrotransposons, and RNA editing in the pig genome was associated with these elements (Huang *et al*. 2021). In our study, we revealed that 84.0% of AIRESs were located within the PRE-1 element family, consistent with previous findings (Adetula *et al*. 2021; Huang *et al*. 2021; Yang *et al*. 2019).

A-to-I RNA editing was catalyzed by the ADAR enzyme family (Heraud-Farlow and Walkley 2020; Larsen and Heide-Jørgensen 2021). In mammals, 3 ADARs exist, namely, ADAR1, ADAR2, and ADAR3. Among them, ADAR1 and ADAR2 were widely expressed and possessed catalytic activity. ADAR1 primarily regulated sites in repetitive regions of the genome, while ADAR2 predominantly regulated sites in nonrepetitive regions, particularly in the brain, and affects adenosine (Freund *et al*. 2020). ADAR3 lacks catalytic activity, but studies suggested that ADAR3 inhibited RNA editing by competitively binding to dsRNA (Nguyen *et al*. 2022). In this study, we found that the ADAR1 gene had lower expression levels in the skeletal muscle and skin, ADAR2 had higher concentration in the brain and lung and the expression level of ADAR3 was relatively higher in tissues such as the brain, pituitary, and thymus (Fig. 2f). Given the expanded range of tissues analyzed in this study, some results align with previous findings (Huntley *et al*. 2016; Yao *et al*. 2019; Zhang *et al*. 2019), while others provide additional insights into the expression of the ADAR genes in other tissues.

SESs of 7 tissues were detected (see *Materials and methods*), 1,563 SESs of skeletal muscle located in 981 genes, and those genes were significantly related to GO terms for muscle cell differentiation and the key genes in this pathway include MYOD1 (Rovito *et al*. 2021), DMD (Herbelet *et al*. 2020), SVIL (Hedberg-Oldfors *et al*. 2020), CAV1 (Flucher 2020), and GPCPD1 (Cikes *et al*. 2024), those genes within intron and 3′UTR region, the result indicated that AIRESs in 3′UTR and intron may play a role in skeletal muscle degeneration/regeneration.

miRNAs were a type of endogenous noncoding RNA that play an important role in gene regulation at the post-transcription level (Shang *et al*. 2023). Studies have shown that RNA-editing events in the 3′UTR can affect the binding of mRNA and miRNA of genes related to muscle development, thereby regulating skeletal muscle development (Zhou *et al*. 2020; Adetula *et al*. 2021). A subset of sites in the 3′UTR was chosen from genes associated with skeletal muscle development. In this study, 61 target miRNAs were predicted through bioinformatics analysis, the result indicated that the interaction of these miRNAs and genes could be regulated by RNA editing events; thereby, the skeletal muscle development in pigs could be influenced. We found that RNA editing in the seed region of the 3′UTR of CFLAR mRNA in miR-708-5p affects miR-708-5p binding with the CFLAR gene. Previous studies showed that miR-708-5p can promote C2C12 cells to end the G1 stage and force them into the differentiation stage, and the homology of miR-708-5p among mice, humans, and pigs was 100% (Ramaswami and Li, 2014). Furthermore, miR-708 participates in the regulation of nNOS (part of the muscle malnutrition protein) expression in human myocytes under conditions of malnutrition (Guilbaud *et al*. 2018).

CFLAR was a cell apoptosis regulator and proliferation inducer, and its antiapoptotic function and overexpression-induced apoptosis activity have been elucidated (Tomaipitinca *et al*. 2021). In this study, we identified an A-to-I RNA editing event in the miR-708-5p seed region of the 3′UTR of CFLAR mRNA. Furthermore, we observed that RNA editing at this site can hinder the binding of miR-708-5p to the 3′UTR of CFLAR mRNA. These findings suggested that RNA editing may participate in regulating the expression of skeletal muscle development-related genes by altering the miRNA seed region sequence in the 3′UTR of CFLAR mRNA.

Due to the limitations of the identification method, which relied solely on RNA-seq data and detected variant sites based on the characteristics of RNA-editing sites using strict filtering criteria, the results may have been biased due to the lack of corresponding DNA data. Therefore, subsequent site identification methods needed further improvement. Since CFLAR and miR-708-5p could influence skeletal muscle development, this study used software prediction and dual-luciferase assays to demonstrate that RNA-editing events could affect the binding of CFLAR and miR-708-5p; however, further experimental validation was needed to determine whether this affected pig skeletal muscle development.

### Conclusions

In this study, we utilized 3,813 RNA-seq datasets, identified a total of 127,927 A-to-I RNA editing sites, and systematically mapped the panoramic RNA-editing atlas across different tissues in pigs. We identified potential RNA-editing sites and genes that may be involved in important processes in skeletal muscle development. In addition, we found that RNA-editing events in the 3′UTR of genes related to muscle development can affect the binding of miRNA. In summary, this study greatly expands our knowledge of RNA editing in pigs and provides candidate targets for molecular breeding.

## Data Availability

The data used for the analysis are from public repositories, and the details of all the data used are available in Supplementary Table S1. Supplemental material is available at https://zenodo.org/records/11077095.
